# The microRNA Expression Signature of Lung Adenocarcinoma Harboring *EGFR* Mutations: Identification of Therapeutic Targets for EGFR-TKI Combination Therapy

**DOI:** 10.3390/ijms27146412

**Published:** 2026-07-19

**Authors:** Takuya Tokunaga, Yuya Tomioka, Aya Harada Takeda, Yuka Ishihara, Ayako Nagata, Mayuko Kato, Takayuki Suetsugu, Keiko Mizuno, Masaya Aoki, Kazuhiro Ueda, Naohiko Seki

**Affiliations:** 1 Department of General Thoracic Surgery, Graduate School of Medical and Dental Sciences, Kagoshima University, Kagoshima 890-8520, Japan; k1933430@kadai.jp (T.T.); k2106983@kadai.jp (A.H.T.); k7007579@kadai.jp (Y.I.); k6651640@kadai.jp (M.A.); k7433286@kadai.jp (K.U.); 2 Department of Pulmonary Medicine, Graduate School of Medical and Dental Sciences, Kagoshima University, Kagoshima 890-8520, Japan; k4829264@kadai.jp (Y.T.); k5090698@kadai.jp (T.S.); keim@m.kufm.kagoshima-u.ac.jp (K.M.); 3 Department of Breast and Thyroid Surgery, Graduate School of Medical and Dental Sciences, Kagoshima University, Kagoshima 890-8520, Japan; k8235150@kadai.jp; 4 Department of Functional Genomics, Graduate School of Medicine, Chiba University, Chiba 260-8670, Japan; mayukokato@chiba-u.jp

**Keywords:** lung adenocarcinoma, *EGFR* mutation, microRNA, *miR-206*, *CDK4*, osimertinib, CDK4/6 inhibitor

## Abstract

Approximately half of Japanese patients with lung adenocarcinoma (LUAD) have epi-dermal growth factor receptor (*EGFR*) gene mutations. Therefore, *EGFR* is the most critical therapeutic target in treating LUAD. This study’s purpose is to explore novel therapeutic targets for LUAD and further improve EGFR inhibitor combination therapy. We created microRNA (miRNA) expression signatures from clinical specimens of LUAD patients harboring *EGFR* gene mutations using RNA sequencing. Based on the miRNA signature, we focused on *miR-206*, which had the most downregulated expression. We searched for its target gene in LUAD cells by facilitating transfection of *miR-206* into the *EGFR* mutant cell line PC9 and searching for genes with suppressed expression. A total of 821 genes were downregulated in PC9 cells. Through molecular classification of these genes, we revealed that 18 genes were closely related to the cell cycle. Among these genes, we focused on cyclin-dependent kinase 4 (*CDK4*) and investigated the synergistic effects of a CDK4/6 inhibitor (abemaciclib or palbociclib) and osimertinib, an EGFR inhibitor in LUAD cells. In vitro and three-dimensional culture analyses showed that combination therapy with a CDK4/6 inhibitor and an EGFR inhibitor synergistically suppressed proliferation of LUAD cells with *EGFR* mutations. Our miRNA-based analysis is an excellent strategy for identifying therapeutic targets for LUAD. Continued analysis will reveal target molecules that enhance the therapeutic effects of EGFR inhibitors.

## 1. Introduction

Lung cancer is the leading cause of cancer-related deaths worldwide. Approximately 2.3 million people are diagnosed with lung cancer each year, and approximately 1.8 million people die from it [1]. Lung cancer is histologically divided into two groups: small-cell lung cancer (SCLC) accounts for 15% of patients and non-small-cell lung cancer (NSCLC) accounts for 85% of patients. Within NSCLC, lung adenocarcinoma (LUAD) is the most common type, accounting for approximately 60% of all cases [2]. Surgical resection is the curative treatment for lung cancer, but many patients are diagnosed at an advanced stage and are not candidates for surgery [3,4].

Advances in molecular diagnostic testing and high-throughput sequencing have enabled the identification of driver gene mutations deeply involved in the molecular pathogenesis, e.g., *EGFR*, *KRAS*, *ALK*, *HER2*, *MET*, *RET*, *BRAF*, and *ROS1* [5]. In NSCLC, epidermal growth factor receptor (*EGFR*) gene mutations are found in 30–40% of Asian patients, including Japanese patients. In particular, *EGFR* gene mutations are confirmed in approximately half of Japanese LUAD patients, making it the most frequently observed driver gene mutation. The frequency of *EGFR* mutations has been reported to be higher in Japanese patients compared to Caucasians [6].

Therefore, *EGFR* is a central target in lung cancer treatment. Since the introduction of the first generation of EGFR tyrosine kinase inhibitors (TKIs), gefitinib and erlotinib, in 1997, new EGFR-TKIs have been continuously developed [7]. While first-generation EGFR-TKIs significantly improved overall survival compared to chemotherapy, resistance was unavoidable and typically developed about 10 months after first-line EGFR-TKI treatment [8,9]. One of the main causes of EGFR-TKI resistance was reported to be the newly occurring T790M mutation in *EGFR* [10].

In 2018, osimertinib was approved as a third-generation EGFR-TKI that was effective against lung cells with the T790M resistance mutation, which did not respond to conventional second-generation EGFR-TKIs [11,12]. However, it was reported that the C797S mutation occurred in approximately 20% of patients using osimertinib, resulting in drug ineffectiveness [13]. Comprehensive genomic analysis of lung cancer cells that have acquired resistance to EGFR-TKIs is extremely important for elucidating the molecular mechanisms of drug resistance. Furthermore, developing EGFR-TKI combination therapies to avoid drug resistance is also an important challenge.

The Human Genome Project revealed that the human genome contains an astonishing number of non-protein-coding RNA molecules, and that these RNA molecules are actually transcribed and functional within cells [14]. Among the various types of non-protein-coding RNAs, microRNAs (miRNAs), which are short, single-stranded RNA molecules, are being studied extensively in cancer research fields. This work uncovered the existence of oncogenic and antitumor miRNAs [15,16]. One of the biological characteristics of miRNAs is that a single miRNA regulates the expression of a very large number of protein-coding genes within a cell [17]. Therefore, aberrantly expressed miRNAs within cancer cells induce breakdown of the tightly ordered RNA networks [18].

First, we created a novel miRNA expression signature using clinical tissue derived from *EGFR* mutation-positive LUAD patients through RNA sequencing. Based on the miRNA signature, we focused on *miR-206*, which had the most downregulated expression in patient tissues. Functional analysis of *miR-206* using the *EGFR* mutant cell line PC9 revealed that *miR-206* expression significantly suppressed the malignant traits of cancer cells [19,20].

Next, therapeutic targets for LUAD were selected from among numerous genes regulated by antitumor *miR-206* within LUAD cells. We performed molecular classification on 821 genes regulated by antitumor *miR-206* and selected 18 genes closely involved in the cell cycle pathway (*CCND1*, *E2F5*, *SMC1A*, *YWHAQ*, *RBL1*, *ORC1*, *MCM6*, *PKMYT1*, *CDK4*, *MCM3*, *BUB1B*, *PRKDC*, *DDX11*, *TICRR*, *CDC7*, *E2F2 OCR6*, and *ATR*). These genes are closely involved in the malignant transformation of LUAD, and exploring the RNA networks regulated by these genes may lead to the discovery of novel therapeutic target molecules.

In this study, we focused on cyclin-dependent kinase 4 (*CDK4*) from among these target genes. CDK4/6 are switches that trigger cell division and proliferation. They work in conjunction with cyclin D to control whether a cell progresses from the G1 phase to the S phase of the cell cycle [21]. CDK4/6 inhibitors (e.g., palbociclib, ribociclib, and abemaciclib) have been developed and are used for the treatment of hormone receptor (HR)-positive and epidermal growth factor receptor 2 (HER2)-negative breast cancer [22]. Furthermore, this study investigated whether the combination of an EGFR inhibitor and a CDK4/6 inhibitor is effective in treating *EGFR* mutation-positive LUAD.

Our analytical strategy, based on *EGFR* mutation-positive LUAD miRNA signatures and miRNA target discovery, will accelerate the search for novel therapeutic target molecules for this disease.

## 2. Results

### 2.1. RNA Sequencing-Based miRNA Expression Signatures for LUAD Patients with EFGR Mutations

To elucidate dysregulated miRNA expression in *EGFR* mutation-positive LUAD cells, we created miRNA expression signature using patient tissues. The clinical information of the patients used for RNA sequencing is shown in Table S1.

In this study, we sequenced 10 small RNA libraries (5 normal lung specimens and 5 lung cancer specimens). The miRNA expression signature was created by comparing the count of miRNA read sequences in normal tissue and cancerous tissue. We identified 30 miRNAs with suppressed expression in cancerous tissue compared to normal tissue (log_2_ Fold change < −1.0 and *p*-value < 0.05; Table 1). Quality-control metrics for the small RNA-seq libraries are summarized in Table S2. All samples yielded sufficient surviving reads after filtering and were used for miRNA quantification.

**Figure 1:**
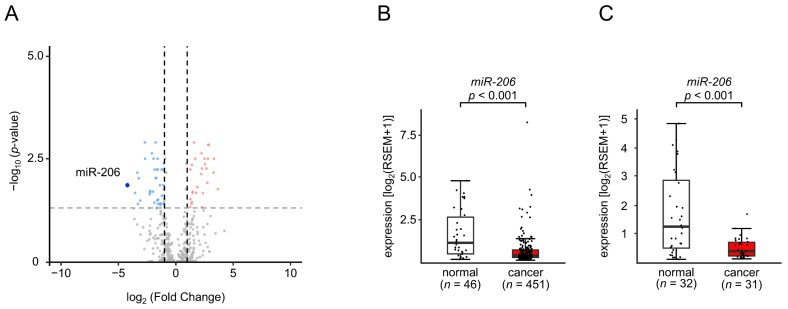
Expression levels of *miR-206* in lung adenocarcinoma (LUAD) clinical specimens. (**A**) Volcano plot of the microRNA (miRNA) expression signature based on miRNA sequencing. The log_2_ fold change (FC) in expression is plotted on the *x*-axis and the log_10_ *p*-value on the *y*-axis. The blue and red dots represent the downregulated (log_2_ FC < −2.0 and *p* < 0.05) and upregulated (log_2_ FC > 2.0 and *p* < 0.05) miRNAs, respectively. (**B**) Expression levels of *miR-206* validated in LUAD clinical specimens (normal lung tissues, *n* = 46; LUAD tissues, *n* = 451). The expression was significantly downregulated in cancerous tissues. (**C**) Expression levels of *miR-206* validated in epidermal growth factor receptor (*EGFR*)-mutant LUAD clinical specimens (normal lung tissues, *n* = 32; *EGFR*-mutant LUAD tissues, *n* = 31). The expression was significantly downregulated in cancerous tissues. Statistical significance was evaluated using the Mann–Whitney *U* test.

This study focused on *miR-206*, which had the most suppressed expression in cancerous tissues, and explored its antitumor functions in *EGFR* mutation-positive lung cancer cells (Table 1 and Figure 1A). We used The Cancer Genome Atlas (TCGA) dataset, a large-scale cohort dataset, to confirm the downregulation of *miR-206* expression indicated by miRNA signatures. Expression levels of *miR-206* were significantly lower in LUAD or LUAD with *EGFR* mutation tissues compared to noncancerous tissues (Figure 1B,C).

### 2.2. Functional Significance of miR-206 in LUAD Cell Lines with EGFR Mutations

**Figure 2:**
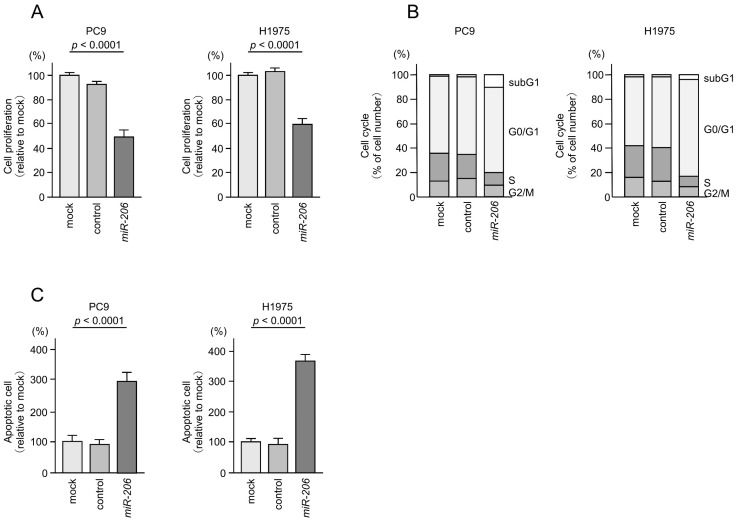
Antitumor functions of *miR-206* in PC9 and H1975 cells. (**A**) Cell proliferation was assessed using XTT assays 72 h after transfection with *miR-206* in PC9 and H1975 cells. (**B**) Cell cycle changes were analyzed using flow cytometry. Assays were performed 72 h after transfection with *miR-206*. The percentages of cells in each cell-cycle phase are shown as the mean values from three independent experiments. (**C**) Apoptotic cells were evaluated using flow cytometry following Annexin V-FITC and PI-PerCP-Cy5-5-A staining 72 h after transfection with *miR-206* in PC9 and H1975 cells. For (**A**,**C**), data are presented as the mean ± SD from at least three independent experiments. Statistical significance was evaluated using one-way ANOVA followed by Tukey’s post hoc test. *p*-values are shown in each panel.

Two cell lines with *EGFR* mutations (PC9 and H1975) were used for the functional analysis of *miR-206*. Ectopic expression of *miR-206* significantly suppressed cancer cell proliferation (Figure 2A). Cell cycle analysis revealed that expression of *miR-206* led to cell accumulation during the G0/G1 phases and an increase in the sub-G1 population (Figure 2B). The apoptosis assay showed that the expression of *miR-206* significantly increased the number of apoptotic cells (Figure 2C).

Furthermore, it was demonstrated that *miR-206* expression significantly suppressed the invasion and migration capabilities of LUAD cells (Figure S1).

These results demonstrate that *miR-206* possesses a potent tumor-suppressing function in *EGFR* mutation-positive cells. In particular, they strongly suggest that *miR-206* regulates the cell cycle and apoptosis.

### 2.3. Identification of Oncogenic Targets of miR-206 Regulation in LUAD Cells with EGFR Mutations

**Figure 3:**
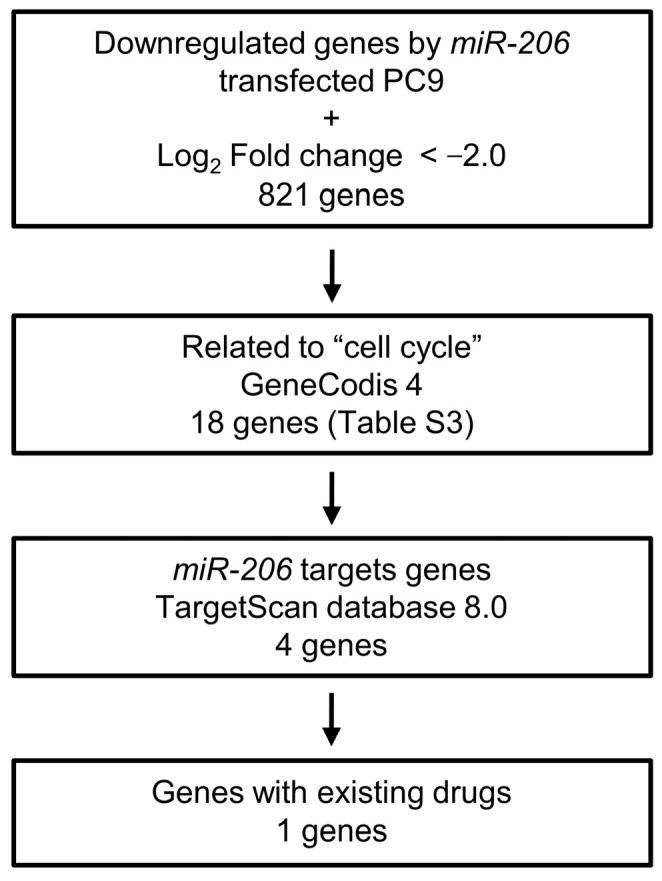
A flowchart for the identification of therapeutic targets regulated by *miR-206* in LUAD with *EGFR* mutations. The characteristics of the 18 cell-cycle-related genes, including fold changes after *miR-206* transfection, predicted *miR-206* binding sites, expression profiles, prognostic relevance, and inhibitor availability, are summarized in Table S3.

Since the tumor-suppressing effects of *miR-206* in LUAD cells with *EGFR* mutations were revealed, we searched for tumor-promoting genes regulated by *miR-206*. Figure 3 shows a flowchart of the *miR-206* target search strategy in LUAD cells with *EGFR* mutations.

First, *miR-206* was transfected into *EGFR*-mutated LUAD cells (PC9), and RNA sequencing was used to identify genes with expression controlled by *miR-206* in PC9 cells. When *miR-206* was ectopically expressed in PC9 cells, the expression of a total of 821 genes was suppressed. Next, we performed an analysis using the GeneCodis4 database to determine which molecular pathway these genes belonged to. The analysis revealed that the genes controlled by *miR-206* belonged to molecular pathways involved in the cell cycle and DNA repair (Table 2).

Based on the functional analysis of *miR-206*, we focused on 18 genes belonging to the cell cycle pathway and narrowed down the therapeutic targets for *EGFR* mutation-positive LUAD.

In this study, *miR-206*-transfected PC9 cells were sequenced. Sequencing revealed that 821 genes were significantly downregulated (Log_2_ FC < −2.0). When these 821 genes were molecularly classified, 18 genes were found to be involved in the cell cycle pathway. qRT-PCR validation confirmed that all 18 genes were significantly downregulated after *miR-206* transfection in both PC9 and H1975 cells (Figures S2 and S3). According to a TargetScan database analysis, 4 of the 18 genes (*CCND1*, *E2F5*, *CDK4*, and *YWHAQ*) contained *miR-206* binding sequences in their 3′ UTR. Of these, cyclin-dependent kinase 4 (*CDK4*) was selected as the target of *miR-206* because it is a predicted direct target and clinically available CDK4/6 inhibitors can be used to pharmacologically target *CDK4*. Other *miR-206*-regulated cell-cycle genes may also be therapeutic candidates and should be investigated in future studies.

### 2.4. Expression and Clinical Features of miR-206 Target Genes (CCND1, E2F5, CDK4, and YWHAQ) in LUAD Cells

**Figure 4:**
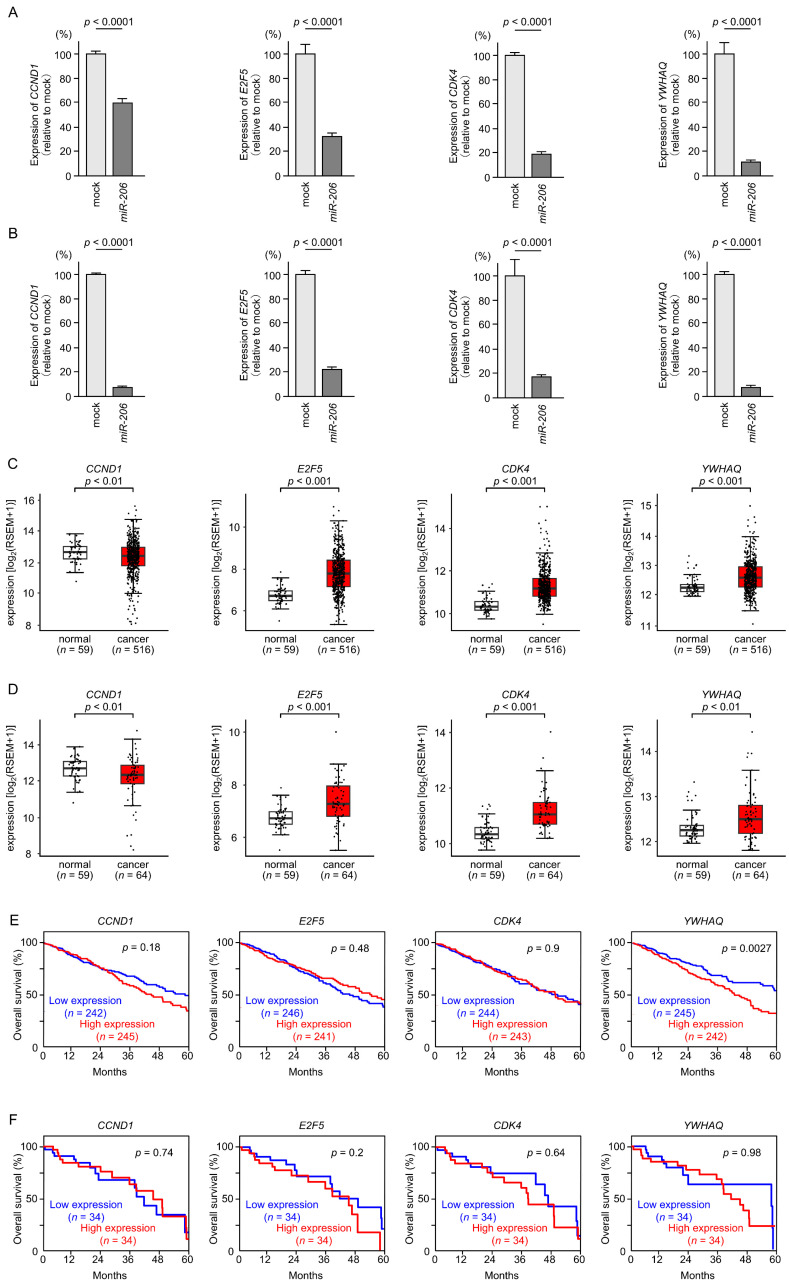
Expression and clinical features of four genes (*CCND1*, *E2F5*, *CDK4*, and *YWHAQ*) regulated by *miR-206* in LUAD cells. (**A**,**B**) Expression of four genes (*CCND1*, *E2F5*, *CDK4*, and *YWHAQ*) was significantly reduced in *miR-206*-transfected PC9 and H1975 cells compared with parental cells. (**C**) The expression levels of four genes were analyzed using The Cancer Genome Atlas (TCGA)-LUAD dataset (normal lung tissues, *n* = 59; LUAD tissues, *n* = 516). (**D**) The expression levels of four genes were analyzed using the *EGFR*-mutated LUAD dataset (normal lung tissues, *n* = 59; *EGFR*-mutant LUAD tissues, *n* = 64). For (**C**,**D**), statistical significance was evaluated using the Mann–Whitney *U* test. (**E**) Kaplan–Meier curves for the 5-year overall survival rate based on the expression of the four genes using the TCGA-LUAD dataset. Patients (*n* = 487) were stratified into high- and low-expression groups based on the median expression level of each gene. The red and blue lines represent the high- and low-expression groups, respectively. (**F**) Kaplan–Meier curves for the 5-year overall survival rate based on the expression of the four genes using the *EGFR*-mutated LUAD dataset (*n* = 68 patients).

Analysis of 18 genes using the TargetScan (Release 8.0) database revealed that four genes (*CCND1*, *E2F5*, *CDK4*, and *YWHAQ*) have *miR-206* binding sequences in their 3′ untranslated region (UTR). Consistent with this, the expression of these four genes was significantly reduced by expression of *miR-206* into PC9 cells (Figure 4A) and H1975 cells (Figure 4B).

We investigated the expression of these four genes in patient tissues and the impact of their expression on prognosis using the TCGA dataset.

We found that expressions levels of the four genes were significantly upregulated in LUAD cancer tissue compared to normal lung tissue (Figure 4C). Even in analyses limited to *EGFR* mutation-positive LUAD tissues, the four genes were highly expressed in cancerous tissue (Figure 4D).

This study examined the effects of four gene expression levels on patient prognosis but found that the expression levels of three genes (*CCND1*, *E2F5*, and *CDK4*) did not adversely affect the prognosis of LUAD patients (Figure 4E,F). *YWHAQ* gene expression was associated with an adverse effect on the prognosis of LUAD patients. However, no such effect was observed in *EGFR* mutation-positive patients (Figure 4E,F). Although *CDK4* expression was not prognostic, *CDK4* remains a therapeutically actionable target downstream of *miR-206* because clinically available CDK4/6 inhibitors can inhibit *CDK4*.

### 2.5. Direct Regulation of CDK4 by miR-206 in LUAD Cells with EGFR Mutations

To verify that *CDK4* is directly regulated by *miR-206*, we investigated *CDK4* expression in *miR-206*-transfected LUAD cells and performed luciferase reporter assays using a vector containing the *miR-206* binding sequence.

**Figure 5:**
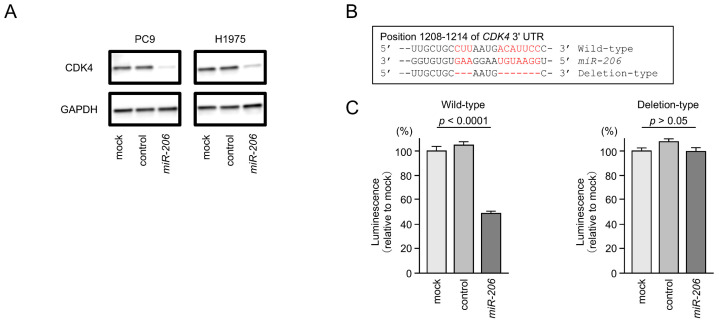
Direct regulation of CDK4 by *miR-206* in *EGFR*-mutated LUAD cells. (**A**) Expression of CDK4 protein levels was markedly reduced in *miR-206*-transfected PC9 and H1975 cells. Protein samples were collected 72 h after *miR-206* transfection and quantified by Western blotting. Glyceraldehyde-3-phosphate dehydrogenase (GAPDH) was used as an internal control. (**B**) The estimated *miR-206* binding site in the 3′ UTR of *CDK4*, as shown by the TargetScanHuman database (release 8.0). Nucleotides shown in red indicate the putative *miR-206*-binding region and the deleted region in the mutant construct. (**C**) Dual-luciferase reporter assays revealing the reduced luminescence activity after co-transfection of *miR-206* with a vector containing the *miR-206* binding site (wild-type) in PC9 cells. In contrast, no such reduction in luminescence activity was observed after transfection with a vector lacking the respective binding sites.

In LUAD cells transduced with *miR-206*, the expression of *CDK4* at the mRNA level was suppressed (Figure 4A,B). At the CDK4 protein level, expression was also markedly reduced in *miR-206*-transfected LUAD cells with *EGFR* mutations (Figure 5A). Full-size images of Western blots are shown in Figure S4.

We also performed luciferase reporter assays to determine whether *miR-206* binds directly to its target sequence within *CDK4*. The target sequence of *miR-206* annotated in the 3′ UTR of *CDK4* is shown in Figure 5B. Suppression of luminescence activity was observed in cells transfected with a vector carrying the wild-type *miR-206* binding sequence of the *CDK4* 3′ UTR (Figure 5C). On the other hand, no suppression of luminescence activity was observed in cells transfected with a vector carrying a deleted *miR-206* binding sequence (Figure 5C).

### 2.6. Efficacy of Osimertinib and CDK4/6 Inhibitors (Abemaciclib and Palbociclib) Combination Therapy in LUAD Cells with EGFR Mutations

CDK4/6 inhibitors block the functions of CDK4 and CDK6, which are kinases that control cell cycle progression. Currently, the CDK4/6 inhibitors used in Japan are abemaciclib and palbociclib.

**Figure 6:**
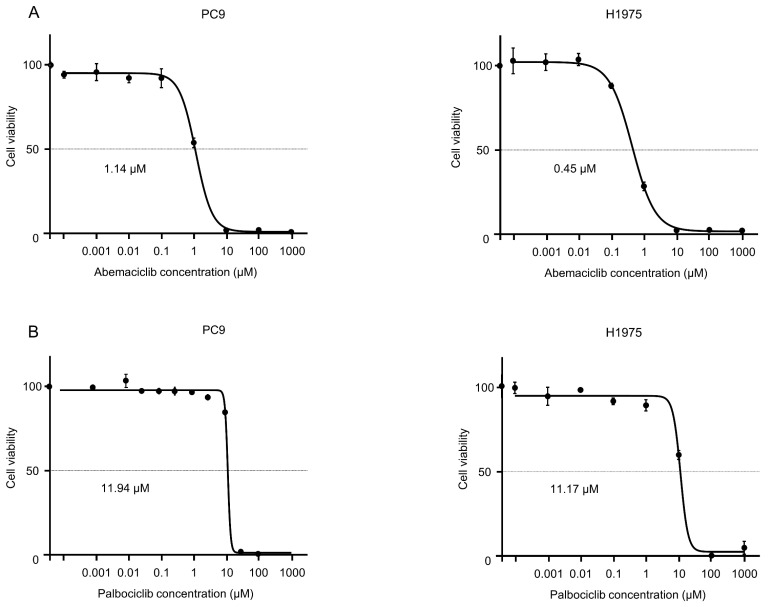
Effect of treatment with CDK4 inhibitors (abemaciclib and palbociclib) on *EGFR*-mutated LUAD cells (PC9 and H1975). The proliferation of PC9 and H1975 cells was inhibited by abemaciclib (**A**) and palbociclib (**B**).

We investigated the effectiveness of combination therapy comprising osimertinib plus a CDK4/6 inhibitor against LUAD cells with *EGFR* mutations (PC9 and H1975). The half-maximal inhibitory concentration (IC_50_) values of abemaciclib were 1.14 µM in PC9 cells and 0.45 µM in H1975 cells, whereas those of palbociclib were 11.94 µM and 11.17 µM, respectively (Figure 6A,B).

**Figure 7:**
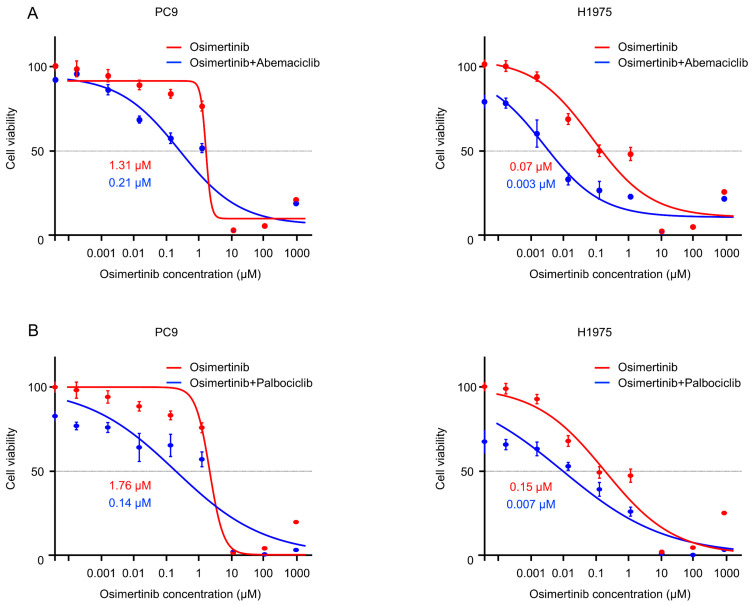
Effect of co-treatment with CDK4 inhibitors and osimertinib in PC9 and H1975 cells. LUAD cells exhibited increased sensitivity to osimertinib when co-treated with the CDK4 inhibitor abemaciclib (0.1µM) (**A**) or palbociclib (1µM) (**B**). Each dot represents the mean cell viability calculated from the XTT assay at each drug concentration, and error bars indicate SD.

Combination treatment with abemaciclib and osimertinib markedly enhanced growth inhibition compared with osimertinib alone. Notably, the IC50 of osimertinib was significantly reduced from 1.31 µM to 0.21 µM in PC9 cells and from 0.07 µM to 0.003 µM in H1975 cells (Figure 7A). Similarly, combination treatment with palbociclib and osimer-tinib also enhanced growth inhibition, reducing the IC50 of osimertinib from 1.76 µM to 0.14 µM in PC9 cells and from 0.15 µM to 0.007 µM in H1975 cells (Figure 7B).

**Figure 8:**
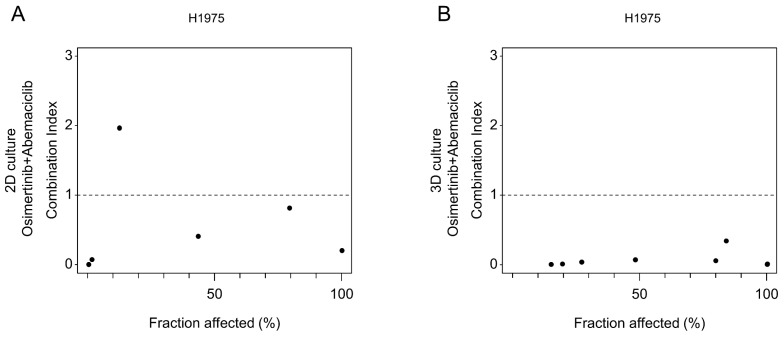
Synergistic effects of osimertinib and abemaciclib in H1975 cells. Synergistic interactions between osimertinib and abemaciclib were evaluated using the Chou–Talalay method. (**A**) Combination index (CI) analysis obtained from two-dimensional (2D) cell culture conditions. (**B**) CI analysis obtained from three-dimensional (3D) cell culture conditions. Osimertinib and abemaciclib were used at a fixed concentration ratio of 1:100 in all experiments.

We investigated the combination index (CI) to see if the combination of osimertinib and abemaciclib showed synergistic effects compared with osimertinib monotherapy. CI analysis revealed strong synergistic effects in H1975 cells, with CI values of 0.28 in two-dimensional (2D) culture and 0.062 in 3D culture at a fractional activity (Fa) of 0.5 (Figure 8). Representative images of the corresponding 3D culture experiments are shown in Figure S5.

These results suggest that the combined inhibition of EGFR and CDK4/6, particularly using abemaciclib, represents a promising therapeutic strategy for *EGFR*-mutant LUAD.

## 3. Discussion

Advances in RNA sequencing technology have made it possible to rapidly and accurately create miRNA expression signatures from a variety of samples. Our research group has so far created miRNA signatures from clinical specimens derived from lung cancer (e.g., small-cell lung cancer autopsy specimens and LUAD brain metastasis tissue) and is attempting to elucidate the oncogenic RNA networks of lung cancer cells starting from antitumor miRNAs [23,24]. In this study, we generated an exploratory miRNA expression signature from *EGFR*-mutant LUAD specimens. We focused on *miR-206*, which had the most suppressed expression in patients’ tissues. To date, *miR-206* has been reported as an antitumor miRNA in various types of cancers [25,26,27]. However, the oncogenic targets and molecular pathways controlled by antitumor *miR-206* vary depending on the type of cancer [20].

In this study, we functionally classified the genes regulated by *miR-206* in *EGFR* mutation-positive cells and selected genes involved in the cell cycle pathway. Starting with antitumor *miR-206*, 18 genes were selected as candidate therapeutic targets for LUAD with *EGFR* mutations. Furthermore, high expression of six genes (*YWHAQ*, *ORC1*, *PKMYT1*, *BUB1B*, *TICRR*, and *ORC6*) was associated with a poor prognosis in patients with LUAD. Furthermore, the expression of these genes may be closely associated with sensitivity to EGFR-TKIs. Cohort studies are warranted to investigate the association between treatment response and the expression status of these genes in patients receiving EGFR-TKI therapy.

Budding uninhibited by benzimidazoles 1 beta (*BUB1B*) was identified as a major regulator of LUAD stemness. Functional analysis showed that *BUB1B* promoted LUAD progression by activating the Ca^2+^/PI3K/AKT signaling pathway [28]. Another study reported that inhibiting *BUB1B* expression increased the sensitivity of LUAD cells to gemcitabine [29].

Serine/threonine kinase cell division cycle 7 (*CDC7*) was identified as a target molecule that showed a synergistic effect with chemotherapy through genome-wide CRISPR/Cas9 screening in SCLC. The CDC7 inhibitor XL413 showed synergistic effects with both cisplatin and etoposide in chemotherapy-resistant SCLC cells [30].

A previous study revealed that suppressing *PKMYT1* expression resolved radiation-induced G2/M phase arrest and reduced the survival rate of irradiated cells. This result suggests that controlling *PKMYT1* may enhance the radio-sensitivity of LUAD cells [31].

Recent studies using the PKMYT1 inhibitor RP-6306 on pancreatic cancer cells reported that RP-6306 effectively suppressed the proliferation of pancreatic ductal adenocarcinoma in vitro and in vivo. Furthermore, its antitumor activity was enhanced and metastasis was suppressed when used in combination with gemcitabine [32].

This study focused on *CDK4*, the direct target molecule of *miR-206*. This is because molecularly targeted therapies against CDK4/6 (e.g., palbociclib, ribociclib, and abemaciclib) have been approved and are being used clinically for breast cancer [33,34,35]. CDK4/6 inhibitors block the transition from the G1 to the S phase of the cell cycle by inhibiting the phosphorylation of retinoblastoma (RB) proteins via the CDK4/6 signaling pathway [21]. CDK4/6 inhibitors are approved and used in patients with HR-positive and HER2-negative metastatic breast cancer as monotherapy, in combination with fulvestrant, or in combination with aromatase inhibitors [22].

This study investigated the efficacy of combination therapy with a third-generation EGFR inhibitor (osimertinib) and a CDK4/6 inhibitor (abemaciclib or palbociclib) using in vitro and three-dimensional (3D) spheroid cell assays. We showed that combining the two inhibitors (osimertinib/abemaciclib and osimertinib/palbociclib) synergistically suppressed the proliferation of *EGFR* mutation-positive LUAD cells. The efficacy of osimertinib in combination with CDK4/6 inhibitors in *EGFR* mutant cells has been reported in previous studies [36,37]. Analysis using osimertinib-resistant cell lines showed that abemaciclib suppressed cancer cell malignant transformation (e.g., proliferation, spheroid formation, and colony formation), but its efficacy was not enhanced in the presence of osimertinib [38]. Analysis using osimertinib-sensitive cell lines showed that the combination of abemaciclib and osimertinib significantly suppressed the development of osimertinib resistance [38]. LUAD cells inevitably develop resistance to EGFR-TKI therapy, and effective treatments for cancer cells that acquire osimertinib resistance have not yet been established [39]. Exploring the molecular oncogenic pathways involved in EGFR-TKI resistance acquisition in LUAD cells could provide clues for therapies that can either reverse resistance or delay the time it takes for resistance to develop.

In this study, we focused on *miR-206* based on the microRNA signatures of *EGFR* mutation-positive patients and identified CDK4/6 inhibitors that showed efficacy in combination with osimertinib through miRNA-based targeting analysis. This study revealed numerous promising LUAD therapeutic targets regulated by *miR-206*. Furthermore, exploring oncogenic pathways regulated by antitumor miRNAs other than *miR-206* in this signature will likely lead to the discovery of numerous therapeutic targets for this disease.

## 4. Materials and Methods

### 4.1. Clinical Specimens and LUAD Cell Lines

Ten clinical tissue samples (five primary cancer tissues and five normal lung tissues) were used to construct miRNA expression signatures for LUAD with *EGFR* mutations. The surgical procedures were performed at Kagoshima University Hospital (between 2017 and 2019), and written consent was obtained from all patients. This research has been approved by the Ethics Committee Epidemiological and related Studies, Sakuragaoka Campus, Kagoshima University (approval number: 250073 eki). Detailed information about the clinical samples used in this analysis is provided in Table S1.

Human *EGFR*-mutant LUAD cell lines (PC9 and H1975) were obtained from RIKEN BRC (Tsukuba, Japan) and the American Type Culture Collection (Manassas, VA, USA). PC9 cells harbor an *EGFR* exon 19 deletion, whereas H1975 cells harbor *EGFR* L858R and T790M mutations. Two cell lines were cultured in RPMI-1640 supplemented with 10% fetal bovine serum at 37 °C in a humidified atmosphere containing 5% CO_2_.

### 4.2. Construction of the miRNA Expression Signature Based on RNA Sequencing

The RNA samples were sequenced using the Illumina NextSeq 500 system (Illumina, San Diego, CA, USA). The resulting sequencing reads were mapped to the human reference genome, and the miRNA reads were annotated using the miRBase database (https://www.mirbase.org, accessed on 17 October 2024). The process of creating miRNA expression signatures from raw sequencing data was described previously [40], and raw data were registered in the Gene Expression Omnibus (GEO; GEO accession number: GSE330633).

### 4.3. Bioinformatics Analysis

The expression levels of miRNAs and their target genes in LUAD tissues were analyzed using publicly available datasets from TCGA (https://www.cancer.gov/tcga, accessed on 17 October 2024), the Genomic Data Commons Data Portal (https://portal.gdc.cancer.gov/, accessed on 17 October 2024), and FireBrowse (http://firebrowse.org/, accessed on 17 October 2024). Overall survival data were obtained from OncoLnc (http://www.oncolnc.org/, downloaded on 17 October 2024) and cBioPortal (https://www.cbioportal.org/, accessed on 17 October 2024).

### 4.4. Ectopic Expression Assays of miR-206 in LUAD Cells with EGFR Mutations

Ectopically expressed *miR-206* precursors were transfected at a final concentration of 10 nM using Opti-MEM (Gibco, Carlsbad, CA, USA) and Lipofectamine RNAiMAX (Invitrogen, Waltham, MA, USA). The transient transfection procedure was described previously [23,41,42]. The reagents used in this experiment are listed in Table S4.

The functional analyses of cell proliferation, migration, and invasion in cancer cells can be found in our previous papers [23,41,42]. Cell cycle distribution and apoptosis were analyzed using a flow cytometer. Detailed protocols for the functional assays were described in our previous studies [23,41,42]. The reagents used in the experiments are listed in Table S4.

### 4.5. Identification of miR-206 Targets in LUAD Cells with EGFR Mutations

To identify oncogenic targets regulated by *miR-206*, we analyzed gene expression data from PC9 cells transfected with *miR-206* (GEO accession number: GSE332750) in combination with TargetScanHuman (https://www.targetscan.org/vert_80/, accessed on 24 May 2025). Differentially expressed genes were identified by comparing *miR-206*-transfected cells with control cells. Candidate target genes were selected based on the overlap between downregulated genes in the expression dataset and predicted targets from TargetScanHuman. Functional annotation of the candidate *miR-206* target genes was performed using GeneCodis4 (https://genecodis.genyo.es/, accessed on 25 July 2025) [43].

### 4.6. Plasmid Construction and Dual-Luciferase Reporter Assays

The following two sequences were cloned into the psiCHECK-2 vector: the wild-type sequence of the 3′ UTRs of *CDK4* and the deletion-type sequence, which lacked the *miR-206* target sites of *CDK4*. The procedures for the transfection and dual-luciferase reporter assays were provided previously [23,41,42].

### 4.7. Anticancer Effects of an EGFR Inhibitor and CDK4/6 Inhibitors in LUAD Cells with EGFR Mutations

To evaluate the anticancer effects of osimertinib, abemaciclib, and palbociclib in LUAD cells, 2,3-bis(2-methoxy-4-nitro-5-sulfophenyl)-2H-tetrazolium-5-carboxanilide (XTT) assays were performed. Cell proliferation in conventional two-dimensional (2D) cultures was assessed using a Cell Proliferation Kit according to the manufacturer’s instructions. LUAD cells were seeded at a density of 2.0 × 10^3^ cells per well in 96-well plates and treated with increasing concentrations of each drug. After incubation, cell viability was measured and dose–response curves were generated.

For three-dimensional (3D) culture experiments, LUAD cells were cultured in Cell-able^®^ plates and treated with the same concentration of each drug. Cell viability was assessed using the CellTiter-Glo^®^ 3D assay according to the manufacturer’s instructions. Dose–response curves were generated, and IC_50_ values were calculated using R (version 4.5.0; R Core Team, Vienna, Austria; https://www.r-project.org, accessed on 20 December 2025). All reagents and materials are listed in Table S4.

### 4.8. Statistical Analysis

All statistical analyses were performed using R (version 4.5.0; R Core Team, Vienna, Austria; https://www.r-project.org, accessed on 20 December 2025). Differences between two groups were analyzed using Student’s *t*-test or the Mann–Whitney *U* test, as appropriate. Comparisons among multiple groups were performed using a one-way analysis of variance (ANOVA) followed by Tukey’s post-hoc test. Survival analyses were conducted using the Kaplan–Meier method, and differences between survival curves were evaluated using the log-rank test. A *p*-value < 0.05 was considered statistically significant.

## 5. Conclusions

We generated a novel miRNA expression signature based on RNA sequencing from LUAD patients with *EGFR* mutations. In this signature, *miR-206* was most downregulated. Furthermore, *miR-206* exhibited tumor-suppressive functions in *EGFR* mutation-positive cells. We searched for genes regulated by *miR-206* and discovered 18 therapeutic targets involved in cell cycle-related pathways. Since *CDK4* is a direct regulatory gene for *miR-206*, its inhibitors, abemaciclib and palbociclib, were selected. Combining osimertinib with a CDK4/6 inhibitor significantly suppressed the proliferation of LUAD cells harboring *EGFR* mutations. This miRNA-based analysis enabled the identification of target molecules exhibiting a synergistic effect with the EGFR inhibitor osimertinib. Our research strategy will contribute to the development of novel treatment regimens for LUAD with *EGFR* mutations.

## Figures and Tables

**Table 1:** Downregulated miRNAs in lung cancer with *EGFR* mutations compared to those in normal lung tissue, identified using RNA sequencing.

MicroRNA	miR Base Accession No.	Guide or Passenger Strand	Chromosomal Location	Log2 Fold Change	*p*-Value	False Discovery Rate
*hsa-miR-206*	MIMAT0000462	Guide strand	6p12.2	−4.22	<0.001	0.01
*hsa-miR-30a-5p*	MIMAT0000087	Guide strand	6q13	−3.55	<0.001	0.02
*hsa-miR-30a-3p*	MIMAT0000088	Passenger strand	6q13	−3.35	<0.001	0.01
*hsa-miR-126-5p*	MIMAT0000444	Passenger strand	9q34.3	−3.30	0.001	0.04
*hsa-miR-584-5p*	MIMAT0003249	Guide strand	5q32	−3.23	<0.001	0.01
*hsa-miR-126-3p*	MIMAT0000445	Guide strand	9q34.3	−2.70	<0.001	<0.01
*hsa-miR-30c-2-3p*	MIMAT0004550	Passenger strand	6q13	−2.68	<0.001	<0.01
*hsa-miR-497-3p*	MIMAT0004768	Passenger strand	17p13.1	−2.28	<0.001	0.02
*hsa-miR-139-5p*	MIMAT0000250	Guide strand	11q13.4	−2.24	<0.001	0.02
*hsa-miR-490-3p*	MIMAT0002806	Guide strand	7q33	−2.24	<0.001	0.02
*hsa-miR-145-5p*	MIMAT0000437	Guide strand	5q32	−2.09	<0.001	<0.01
*hsa-miR-218-5p*	MIMAT0000275	Guide strand	4p15.31	−1.99	<0.001	0.02
*hsa-miR-143-3p*	MIMAT0000435	Guide strand	5q32	−1.84	<0.001	0.01
*hsa-miR-195-5p*	MIMAT0000461	Guide strand	17p13.1	−1.76	<0.001	0.01
*hsa-miR-145-3p*	MIMAT0004601	Passenger strand	5q32	−1.75	<0.001	<0.01
*hsa-miR-99a-5p*	MIMAT0000097	Guide strand	21q21.1	−1.69	<0.001	0.01
*hsa-miR-223-3p*	MIMAT0000280	Guide strand	Xq12	−1.66	0.001	0.05
*hsa-miR-320e*	MIMAT0015072	Guide strand	19q13.32	−1.59	<0.001	<0.01
*hsa-miR-223-5p*	MIMAT0004570	Passenger strand	Xq12	−1.54	0.001	0.04
*hsa-miR-195-3p*	MIMAT0004615	Passenger strand	17p13.1	−1.54	<0.001	0.03
*hsa-miR-140-5p*	MIMAT0000431	Passenger strand	16q22.1	−1.39	<0.001	0.01
*hsa-miR-99a-3p*	MIMAT0004511	Passenger strand	21q21.1	−1.35	<0.001	0.04
*hsa-miR-140-3p*	MIMAT0004597	Guide strand	16q22.1	−1.34	0.001	0.04
*hsa-miR-130a-3p*	MIMAT0000425	Guide strand	11q12.1	−1.33	<0.001	0.04
*hsa-miR-26a-5p*	MIMAT0000082	Guide strand	3p22.2	−1.21	<0.001	0.03
*hsa-miR-30c-5p*	MIMAT0000244	Guide strand	6q13	−1.09	<0.001	0.01
*hsa-miR-23a-3p*	MIMAT0000078	Guide strand	19p13.12	−1.09	<0.001	0.01
*hsa-miR-30b-3p*	MIMAT0004589	Passenger strand	8q24.22	−1.09	<0.001	0.04
*hsa-miR-143-5p*	MIMAT0004599	Passenger strand	5q32	−1.04	<0.001	<0.01
*hsa-let-7b-5p*	MIMAT0000063	Guide strand	22q13.31	−1.03	<0.001	0.02

**Table 2:** Significantly enriched annotations of downregulated genes after *miR-206* transfection.

Description	*p*-Value	False Discovery Rate	Enrichment	Genes
Homologous recombination	<0.001	<0.001	6.42	*BRCA1*, *RBBP8*, *PALB2*, *RAD54L*, *EME1*, *POLD3*, *BRCA2*, *RAD51D*, *RAD54B*, *XRCC2*, *POLD1*, *RAD51*
DNA replication	<0.001	0.003	5.48	*POLE2*, *MCM6*, *POLD3*, *POLE*, *MCM3*, *PRIM1*, *LIG1*, *POLD1*, *POLA1*
Fanconi anemia pathway	<0.001	0.003	4.55	*MLH1*, *RMI1*, *FANCD2*, *BRCA1*, *PALB2*, *FANCE*, *EME1*, *BRCA2*, *FANCC*, *RAD51*, *ATR*
Cell cycle	<0.001	0.024	2.50	*CCND1*, *E2F5*, *SMC1A*, *YWHAQ*, *RBL1*, *ORC1*, *MCM6*, *PKMYT1*, *CDK4*, *MCM3*, *BUB1B*, *PRKDC*, *DDX11*, *TICRR*, *CDC7*, *E2F2*, *ORC6*, *ATR*
Bladder cancer	<0.001	0.024	4.28	*CCND1*, *THBS1*, *SRC*, *DAPK1*, *FGFR3*, *CDK4*, *E2F2*, *EGFR*
Mismatch repair	<0.001	0.024	5.72	*MLH1*, *MSH6*, *POLD3*, *LIG1*, *POLD1*, *EXO1*
Purine metabolism	0.002	0.092	2.40	*PDE9A*, *HPRT1*, *PDE2A*, *PDE4A*, *ADCY7*, *PNP*, *PAPSS2*, *XDH*, *PGM2*, *AK4*, *PRPS2*, *ENTPD2*, *IMPDH1*, *NT5M*
Pentose phosphate pathway	0.002	0.096	4.24	*G6PD*, *PGM2*, *PRPS2*, *PGD*, *SHPK*, *FBP1*
Base excision repair	0.003	0.117	3.49	*POLE2*, *UNG*, *POLD3*, *POLE*, *LIG1*, *POLD1*, *MUTYH*
Rap1 signaling pathway	0.004	0.117	1.97	*MAPK13*, *SIPA1L3*, *ID1*, *ADCY7*, *CSF1*, *CALML4*, *FGFR4*, *THBS1*, *SRC*, *RGS14*, *LPAR1*, *TLN2*, *SIPA1*, *VEGFC*, *FGFR3*, *ARAP3*, *PRKD3*, *EGFR*, *PLCE1*
Other types of O-glycan biosynthesis	0.005	0.146	3.27	*GALNT10*, *ST6GAL1*, *B3GLCT*, *MFNG*, *GALNT9*, *LFNG*, *GALNT14*
Glycolysis/Gluconeogenesis	0.011	0.286	2.62	*ALDH3A1*, *HKDC1*, *PGAM4*, *ADPGK*, *PGAM1*, *PGM2*, *LDHA*, *FBP1*
Endocrine resistance	0.012	0.311	2.26	*MAPK13*, *CCND1*, *ADCY7*, *NOTCH3*, *SRC*, *NOTCH1*, *NOTCH2*, *CDK4*, *E2F2*, *EGFR*
Pathways in cancer	0.018	0.408	1.44	*MECOM*, *MLH1*, *CCND1*, *PLEKHG5*, *MSH6*, *ADCY7*, *ARNT2*, *TCF7*, *CALML4*, *FGFR4*, *NOTCH3*, *FN1*, *LPAR1*, *DAPK1*, *GNB1*, *TRAF5*, *LAMA4*, *GLI2*, *VEGFC*, *GNA12*, *GNG7*, *NOTCH1*, *ITGA6*, *WNT7A*, *FGFR3*, *BMP4*, *NOTCH2*, *BRCA2*, *CDK4*, *E2F2*, *TPM3*, *EML4*, *RAD51*, *EGFR*, *PPARG*
ECM–receptor interaction	0.020	0.419	2.22	*FN1*, *THBS1*, *ITGB5*, *LAMA4*, *ITGA6*, *CD44*, *SPP1*, *COL6A2*, *SDC1*
Metabolic pathways	0.028	0.553	1.21	*MECOM*, *PDE9A*, *KHK*, *PEMT*, *HPRT1*, *ALDH3A1*, *ACOX2*, *HKDC1*, *FAM213B*, *PDE2A*, *PDE4A*, *PGAM4*, *ADCY7*, *PNP*, *B4GALNT4*, *MGAT5B*, *B4GALT6*, *MGAT5*, *GALNT10*, *CA12*, *GUSB*, *PAPSS2*, *PSAT1*, *ST6GAL1*, *OAT*, *HACD3*, *ATP6V1A*, *SPHK1*, *B3GALNT1*, *XDH*, *EHMT2*, *GALNT9*, *ADPGK*, *AKR1B10*, *UGT1A4*, *AGMAT*, *UGT8*, *XYLT2*, *ADO*, *GK*, *PGAM1*, *G6PD*, *ITPKA*, *MBOAT1*, *HSD3B7*, *HSD11B2*, *CYP2S1*, *ANPEP*, *HADH*, *OXCT1*, *PGM2*, *AK4*, *PLA2G7*, *MOCOS*, *PTGIS*, *GPAT2*, *CHSY1*, *CMBL*, *PRPS2*, *FUT9*, *DGKG*, *PGD*, *SHPK*, *CAD*, *AADAT*, *PLA2G6*, *B4GALNT1*, *GALNT14*, *RENBP*, *SARDH*, *LDHA*, *ENTPD2*, *DOLPP1*, *IMPDH1*, *AMDHD1*, *ACOT7*, *SMOX*, *NT5M*, *LPCAT2*, *ITPKB*, *FBP1*, *MAN2A1*, *NMNAT2*, *PLCE1*, *TK2*
Human papillomavirus infection	0.029	0.553	1.52	*CCND1*, *TCF7*, *NOTCH3*, *FN1*, *THBS1*, *ITGB5*, *ATP6V1A*, *RBL1*, *MFNG*, *LAMA4*, *LFNG*, *NOTCH1*, *ITGA6*, *WNT7A*, *NOTCH2*, *CDK4*, *SLC9A3R1*, *DLG3*, *PPP2R2C*, *SPP1*, *COL6A2*, *EGFR*, *ATR*
Nucleotide metabolism	0.039	0.673	2.06	*HPRT1*, *PNP*, *XDH*, *AK4*, *ENTPD2*, *IMPDH1*, *NT5M*, *TK2*
MicroRNAs in cancer	0.040	0.673	1.61	*CCND1*, *BRCA1*, *NOTCH3*, *THBS1*, *TIMP3*, *SLC45A3*, *HMGA2*, *MARCKS*, *NOTCH1*, *SERPINB5*, *FGFR3*, *NOTCH2*, *HDAC4*, *CD44*, *E2F2*, *EGFR*
Central carbon metabolism in cancer	0.042	0.673	2.16	*HKDC1*, *PGAM4*, *PGAM1*, *G6PD*, *FGFR3*, *LDHA*, *EGFR*

## Data Availability

Publicly available datasets were analyzed in this study. These data can be accessed here: https://www.ncbi.nlm.nih.gov/geo/query/acc.cgi?acc=GSE330633 (accessed on 1 July 2026) and https://www.ncbi.nlm.nih.gov/geo/query/acc.cgi?acc=GSE332750 (accessed on 1 July 2026).

## Supplementary Materials

The following supporting information can be downloaded at: https://www.mdpi.com/article/10.3390/ijms27146412/s1.
