# Supplementary figures and images for: The microRNA Expression Signature of Lung Adenocarcinoma Harboring EGFR Mutations: Identification of Therapeutic Targets for EGFR-TKI Combination Therapy
Source: Int J Mol Sci. 2026 Jul 19;27(14):6412. doi: 10.3390/ijms27146412 (PMC13411351; doi:10.3390/ijms27146412)

## Slide 1
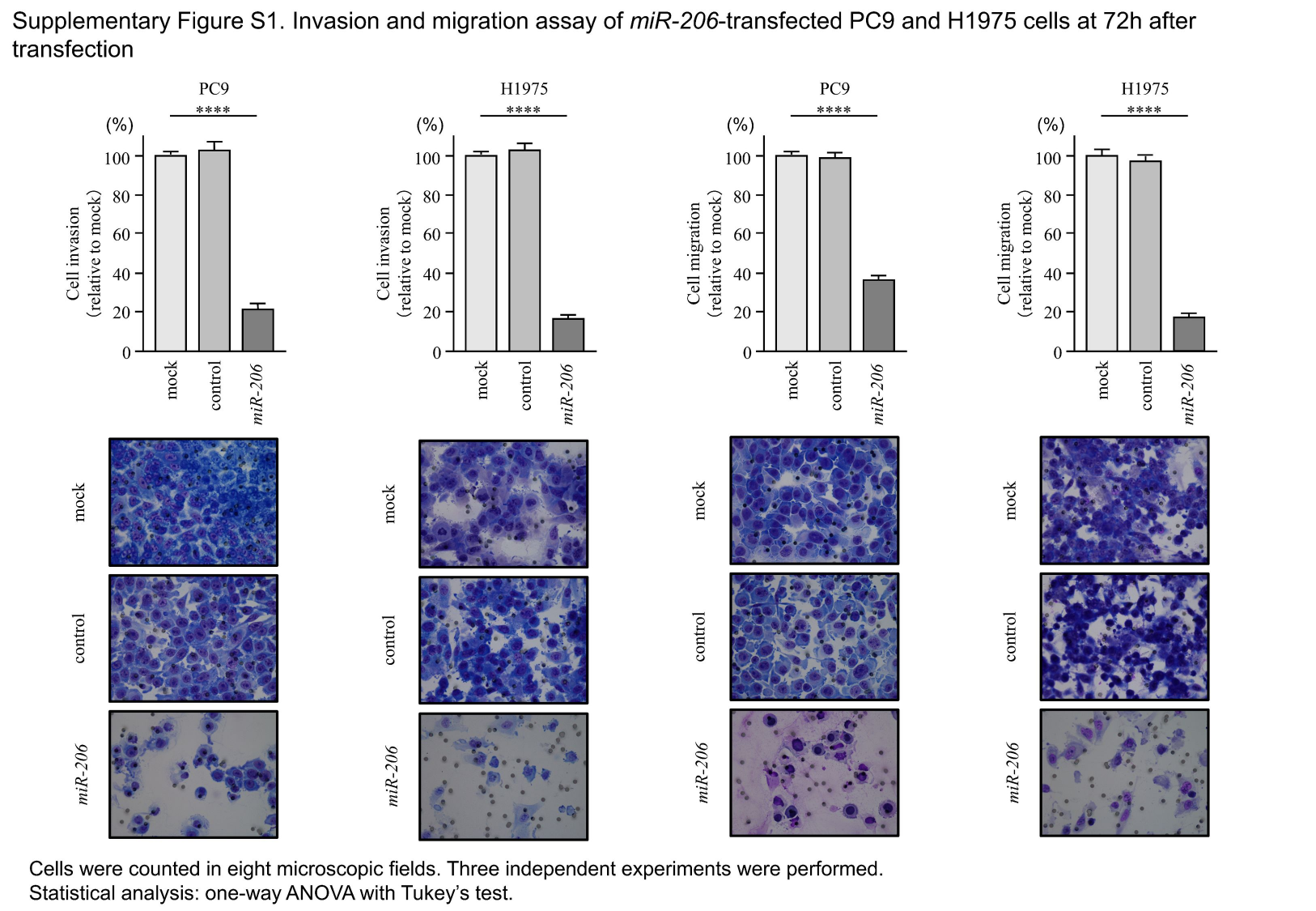

## Slide 2
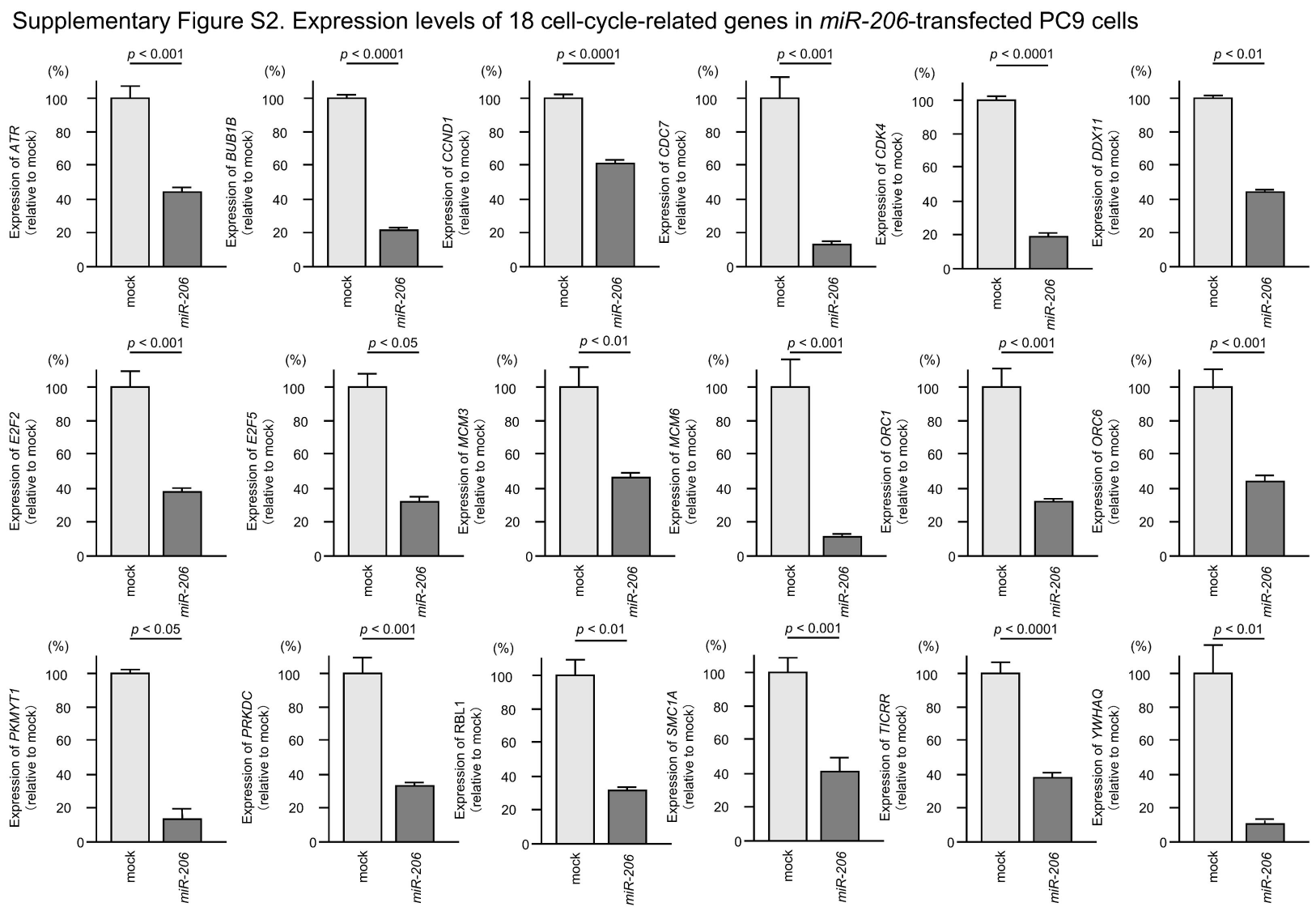

## Slide 3
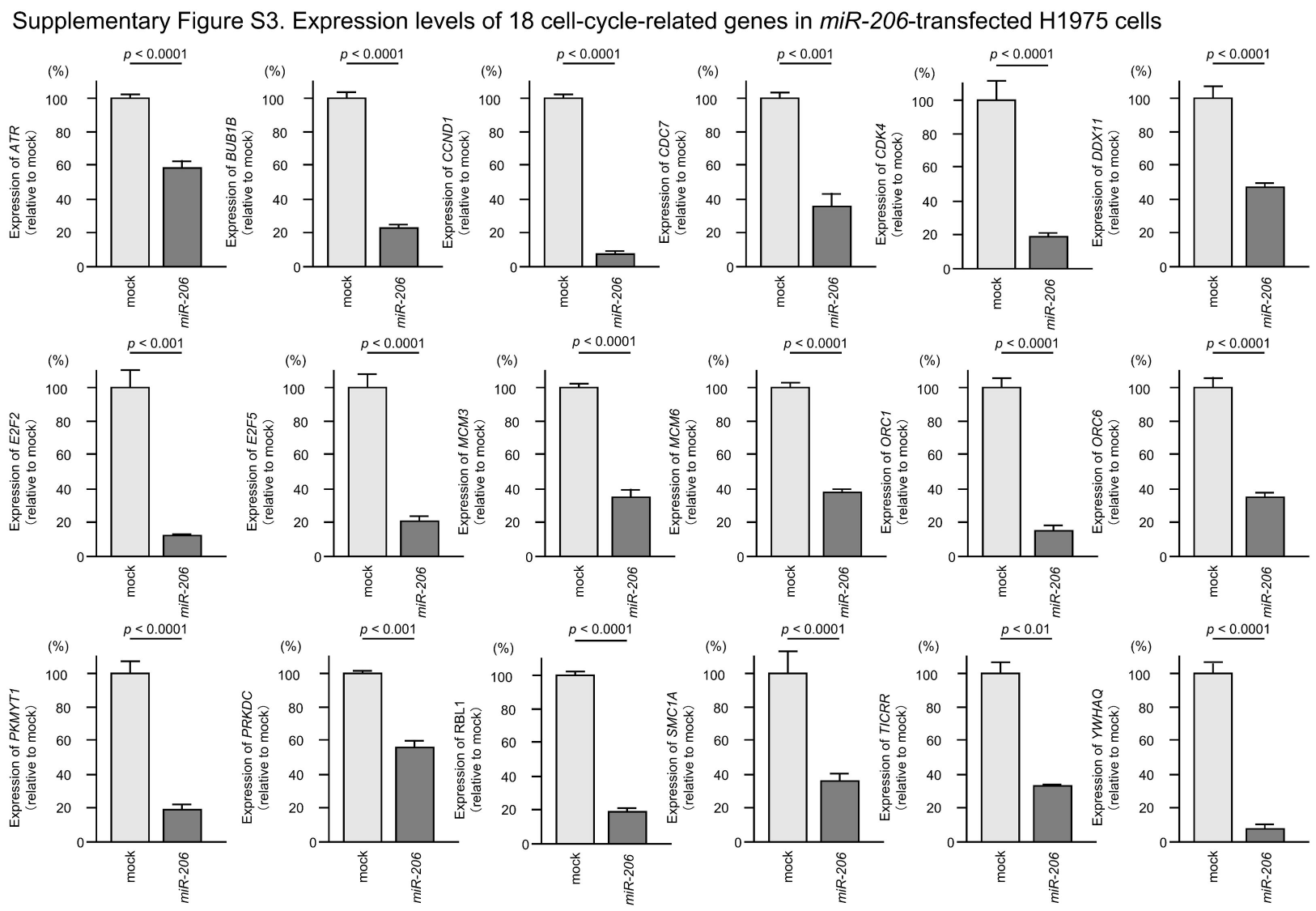

## Slide 4
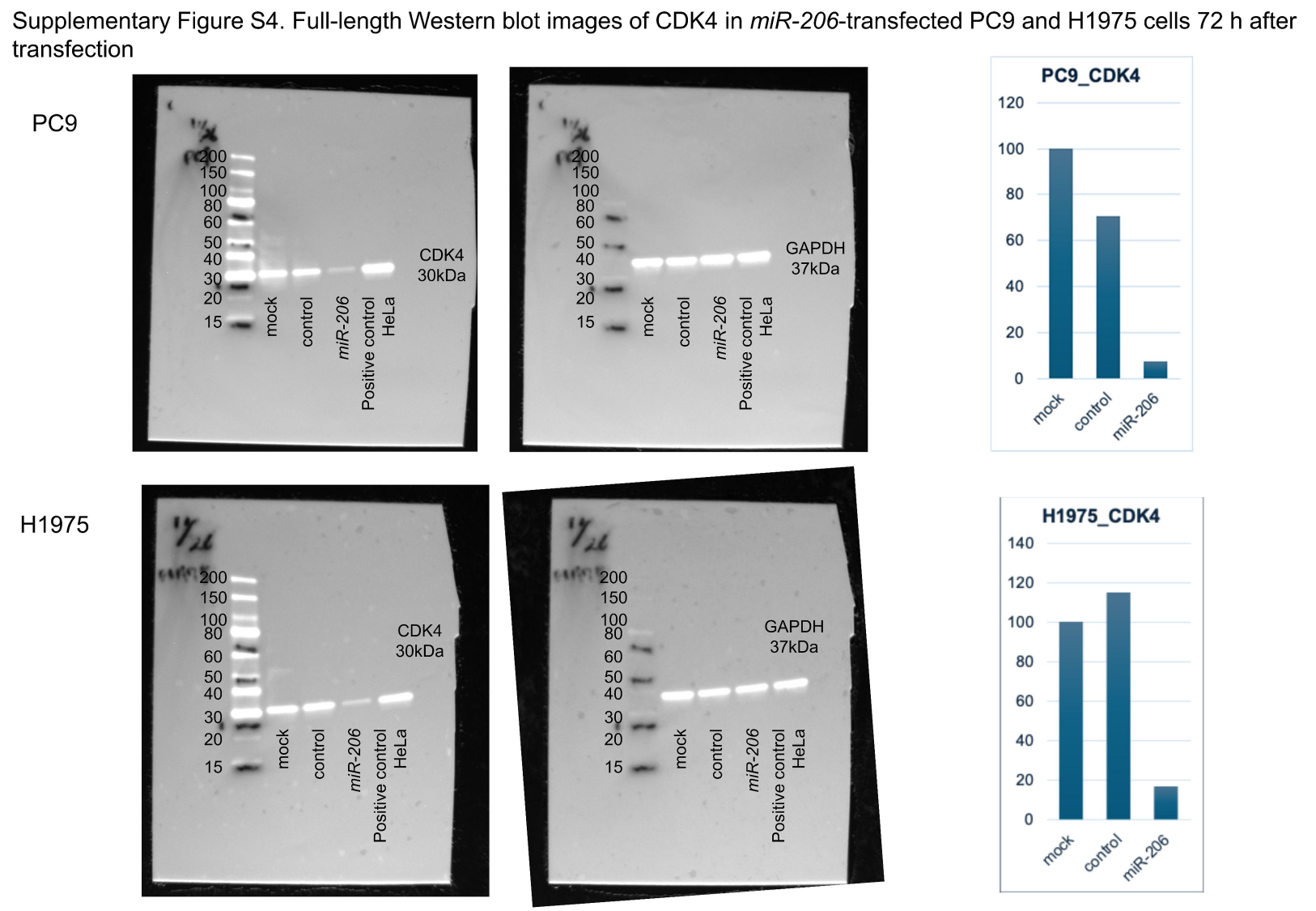

## Slide 5
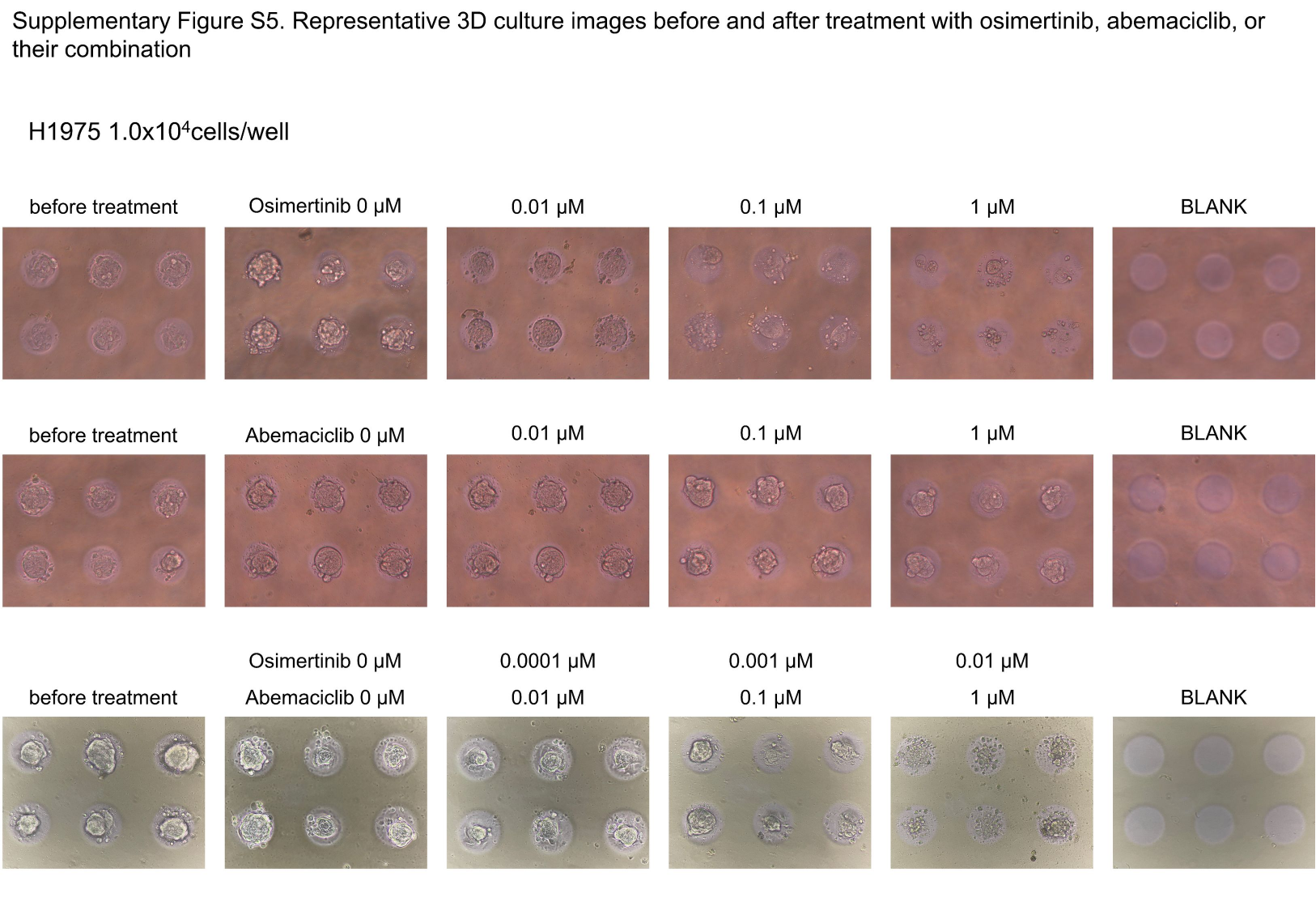

Supplement: Supplementary file 1 [file ijms-27-06412-s001.zip › Supplementary Figure.pptx]
